# Procreative consciousness in a global market: gay men's paths to surrogacy in the USA

**DOI:** 10.1016/j.rbms.2019.03.001

**Published:** 2019-03-21

**Authors:** Marcin Smietana

**Affiliations:** Reproductive Sociology Research Group, University of Cambridge, Cambridge, UK

**Keywords:** gay fathers, LGBTQ reproduction, surrogacy, procreative consciousness, reproductive decision-making, fertility industry

## Abstract

This article explores one of the contemporary contexts of reproductive decision-making: gay men's paths to surrogacy within the globalised USA fertility industry. The stories collected from qualitative interviews and ethnographic research with 37 gay men from several countries in Europe and the USA, who all had children through surrogacy in the USA, show that the men's understandings of their own reproductive aspirations and opportunities changed over time, as if recovering the fertility that was lost by coming out. This shift in the men's procreative consciousness – i.e. in their awareness of being subjects that could reproduce (or not) – disrupts the heteronormative idea that to be queer is not to contribute to the reproduction of the species, the family and the nation. Alongside this consciousness shift, however, reproductive decision-making of the gay men in this study was contingent on multiple factors: access to the fertility industry; economics, given how expensive and thus stratified surrogacy is; social support in the men's communities and extended families; their emotions and values. Therefore these gay men's reproductive decision-making could be characterized in terms of reproductive contingency and consciousness change, within which the globalised fertility industry was one relevant element among the choreography of multiple factors. These findings evidence that despite naturalization of reproduction as an obvious or ‘natural’ event in life, it is contingent, anything but obvious, and its perceptions are changeable. Reproduction is achieved not merely as a result of rational decision-making but rather in the interplay with an array of factors.

## Introduction

On a Sunday morning in San Francisco, over 200 men, mostly in their late 20s to late 40s, listened to the Powerpoint-accompanied talk ‘Plan your surrogacy journey’. They gathered at a surrogacy fair, where agencies, clinics and associations presented their surrogacy offer and advice. In between the talks and presentations that surrogates, gay fathers, professionals, activists and academics gave on interpersonal, legal, medical and other decisions involved in surrogacy, the men browsed the stands of agencies and clinics. The event hallway buzzed with intended fathers seeking information and chatting to agents, physicians, surrogates, egg donors, and activists. Jack and Tom, whom I met at the fair, exchanged business cards and leaflets with an agency they would possibly hire to find a surrogate and an egg donor. In turn, Donna, an experienced and activist surrogate who had given a talk in one of the panels, through informal networking during the event drinks met a gay couple with whom she was now going to undertake a surrogacy arrangement. Some others had not yet taken decisions by the end of the day: Jack, a man in his late 20s, with whom I chatted over nibbles, concluded that he felt a bit overwhelmed and he needed to think it all over and research more. (Field notes, 2016).

This vignette from the research study I carried out with gay men in the process of surrogacy in the USA shows just one of several aspects of their reproductive decision-making, discussed throughout this article. Events such as this surrogacy fair, as well as their online alternatives, first appeared just under a decade ago (see e.g. Men Having Babies, 2018a). Until recently in Euro-American cultures, coming out as gay^1^ to oneself and others often implied a reproductive loss or a non-conventional life course without having children, who did not just come ‘naturally’ conceived at home (Smietana et al., 2014, Stacey, 2006, Weeks, 2018). Yet for 2 decades now, gay partnership and marriage law lobbyists, rainbow family activists, individuals setting up donor and surrogacy arrangements, as well as commercial fertility providers have been gradually contributing to ‘queering reproduction’ (Mamo, 2007) and to the emergence of a new collective ‘procreative consciousness’ (Berkowitz, 2007) of LGBTQ + people's reproductive aspirations. Intentional gay father and lesbian mother families have been created by people who first came to identify as non-heterosexual and only later decided to have children. Even though for many young non-heterosexual people it is still far from obvious if they are going to have children or not and how, in the context of gay marriage, e.g. in the UK, they no longer exclude parenthood from the horizon of their life options (Pralat, 2016, Pralat, 2018).

This may form part of a larger normalization process taking place in several Western countries, within which younger people in formalised same-sex relationships have ‘actively modelled their relationships on a concept of the ordinary rather than the radically different’, as compared with previous generations of lesbians and gay men, for example in the UK (Heaphy, Smart & Einarsdottir 2013: 14), the USA (Berkowitz, 2007, Lewin, 2009) and several European countries such as Spain (Pichardo, 2011).

Following the rise in reproduction by lesbian women, as well as biomedicalization of lesbian family-making practices (Mamo, 2007, Nordqvist, 2012), the last decade has brought a steady increase in cases of intentional gay father families through surrogacy, both in the USA (Gamson, 2015, Goodfellow, 2015, Lewin, 2009), and in other jurisdictions whose citizens travel abroad for surrogacy (see, e.g., Carone et al., 2016 for Italy; Courduriès, 2016 for France; Gamble, 2016 and Golombok, 2015 for the UK; Murphy, 2015 and Riggs, 2015 for Australia; Nebeling Petersen, 2018 for Denmark; Smietana, 2017b for Spain). Gay fathers may constitute well under 50% of all intended parents through surrogacy alongside a majority of heterosexual parents (e.g. see Norton et al., 2015 for the UK). The social and legal relevance of a man's genetic link to the child usually established in surrogacy, as well as pre-birth parental orders given to intended parents in some countries, may attract to commercial surrogacy in the USA those gay men who can afford to spend approximately $150,000 on a surrogacy arrangement. Currently, some states in America offer the only stable market for commercial surrogacy available to gay men worldwide, after the bans on transnational surrogacy in India, Thailand and Mexico, as well as its formal availability in Russia only to heterosexual married couples (for more details see e.g., Jadva, 2016, Thompson, 2016, Smietana et al., 2018, Weis, 2018).

In this market, surrogacy is expensive and thus highly stratified (also see Jacobson, 2018, this volume), even though some people may find ways to make it affordable (e.g., taking mortgage and other loans or selling a house). Stratification underpins surrogacy – whether by economic status in case of commercial surrogacy (e.g. in the USA due to the prohibitive cost of surrogacy), or by citizenship status in case of altruistic arrangements (e.g. in the UK where permanent residents can access surrogacy). Nevertheless, being able to afford commercial surrogacy is neither a straightforward phenomenon itself nor by any means enough for surrogacy to be pursued.

In this article I explore the ways in which the normalization of queer families is taking place on the global surrogacy market. In particular, I examine the process of reproductive decision-making of 37 gay men who became fathers through surrogacy in the USA. The questions that drive this enquiry are why and how some gay men get to embark on complex and costly surrogacy arrangements. What does having children mean to them? How do they opt for surrogacy, and why do some of them travel to the USA for this reason? In what ways do their accounts feature a newly emerging context of reproductive decision-making: the globalised fertility industry^2^?

### Procreative consciousness on reproductive markets

In this article, I use the term reproductive decision-making in a sense akin to reproductive navigation (Van der Sijpt, 2014: 287) taking place among multiple factors that influence people's fertility aspirations and behaviours (and that may include, in particular, sociality, corporeality, as well as power), as individuals ‘constantly reconfigure their choices’ with regard to their social environments. A related approach has also been termed flexible decision-making (Taragin-Zeller, 2019), whereby those who decide often accommodate apparently contradictory aspirations in ways that escape narrower definitions of planned versus unplanned parenthood. Among circumstances on which reproduction is contingent are included structural hierarchies, through which individuals' ‘reproductive choices’ are stratified so only some can make choices of certain kinds (Colen, 1995, Luna and Luker, 2013). It is in this dynamic and interactive context that I use the term reproductive decision-making to refer to my interviewees' navigation among reproductive options, barriers and other multiple factors at play, as well as their own their aspirations, bodies, and changing perceptions and consciousness.

I also refer to a more specific concept of procreative consciousness (Berkowitz, 2007, Berkowitz and Marsiglio, 2007), which concerns, in this case, individuals' ‘self-awareness of being both gay and capable of creating and/or fathering human life’. This type of consciousness is developed by individuals as part of their reproductive decision-making; however, it also emerges at a collective level. Gay men's procreative consciousness must be understood in a historical context in which gay people were excluded from reproduction and from the social power that it endowed (Weeks, 2018), as it is only over the last two decades that coming out as gay has ceased to be considered contradictory to developing reproductive aspirations in a growing number of locations. Hence the concept of gay men's procreative consciousness was used to describe the reproductive aspirations of people who were until recently denied the possibility of having such aspirations; it grasps a change in perceptions and consciousness both for individuals and entire societies, and it brings queerness within the reproductive realm previously demarcated only by heteronormativity.

This study also builds on previous research on reproductive decision-making, which in the context of the USA showed how contingent reproduction was, particularly for gay men, most of whom were found to be ‘situational parents’ (Stacey, 2006). Reproductive contingency was also found to be the case for heterosexual men in the USA, UK, Australia and South Africa (Bartholomaeus and Riggs, 2017, Blell, 2018, Marsiglio, 1991, Morison, 2013), as well as for heterosexual couples in Spain (Alvarez, 2018) – only half of the latter wondered when to have children, whilst the other half wondered whether to actually have children at all. This reproductive contingency could be attributed to different factors that may deter individuals from parenthood: postmodern values of self-fulfilment (Giddens, 1992); pressures on ‘intensive parenting’ (Faircloth and Gurtin, 2017); poor child-care services in the neoliberal context (Briggs, 2017), to which in case of gay men are added increased practical, symbolic and other difficulties to actually achieve parenthood.

Among these multiple elements that underpin reproductive decisions, in the present study I pay particular attention to how the market in reproductive technologies factors in gay men's reproductive decision-making. The assisted reproductive technology (ART) market was found to play a role in women's decisions to postpone parenthood through egg freezing in the UK (Baldwin, 2017). The influence of fertility industries on shaping dominant discourses of fertility has been discussed by Lucy van de Wiel (Van de Wiel, 2018, Van de Wiel, 2019) in case of egg freezing in the UK, the USA and the Netherlands: fertility can now be extended in time thanks to egg freezing, so it is perceived in new ways. The role played by commercial fertility clinics in individuals' perceptions of their own reproductivity was also shown for lesbian women in the USA (Mamo, 2007), and it was noted for gay men in Australia (Murphy, 2013, Murphy, 2015). In what ways then does the global fertility industry feature in gay men's reproductive consciousness, in the individual and collective shifts in this consciousness, and in gay men's decision-making?

## Material and methods

This article stems from a research project on the experiences of surrogacy in the USA.^3^ I carried out the fieldwork discussed here in California and Oregon, where commercial as well as transnational surrogacy is legal and well-established. During the 18 months of fieldwork between 2014 and 2016, I interviewed 37 intended gay fathers forming 20 families (corresponding to 17 couples and 3 single fathers). One half of them (i.e., 10 families) lived permanently in the west, north and south of Europe^4^ yet they travelled to the USA to carry out surrogacy, 2 couples were from Australia and New Zealand, and 8 families were USA citizens. The men who travelled to the USA for surrogacy came from countries where commercial surrogacy was not legal: Denmark, France, Germany, Italy, the Netherlands, Spain, Switzerland and the UK. The majority of the American fathers I spoke to resided permanently in California. Most of the families were in the process of surrogacy during the research so I could follow them through multiple interviews, emails, and participant observation episodes. The men were all aged between 35 and 50 years. Among the 37 intended fathers I spoke to, 29 self-reported their ethnic background as white, four described themselves as South Asian, one as Japanese American (American of Japanese descent) and three self-identified as Latino. All but two of the 37 men had higher education diplomas (30 Master's degrees, four BA degrees, two college Associate degrees, and one PhD). The jobs they carried out were mostly managerial and highly skilled (e.g., a lawyer, an executive director, a physician, a software programmer), except for a few men in other middle-class occupations (e.g. a clerk or a high-school teacher). The broader study also included 20 surrogates and 15 professionals such as attorneys, physicians, agency workers (for further methodological and other details see Smietana, 2017a).

The study was advertised on surrogacy and LGBTQ + community forums, as well as by direct email to all surrogacy agencies and clinics in California. All the research was carried out with the participants' informed consent.^5^ It involved at least one semi-structured in-depth interview with each participant, audio-recorded and later transcribed. Each formal interview started with a general question ‘How did you get to do surrogacy?’ which usually elicited the interviewees' own stories about their pathways to parenthood. Then more detailed questions were asked about several aspects of the men's ‘surrogacy journeys’. The study also included participant observation episodes at public surrogacy events in San Francisco, Los Angeles, New York, Brussels and London; accompanying some of the interviewees to clinic checks, agency meetings, and other surrogacy-related sites on the west coast of the USA; as well as regular email exchanges.

The interview transcripts and other field notes were analyzed thematically, with the aid of ATLAS.ti software. In the process of open coding of the transcripts and field notes, thematic sections were identified and coded first into broad themes and then into subthemes. The main transversal themes from the thematic analysis provided the section titles set out below in this article. At the same time, case study analysis was carried out for each family with the help of written memos, so as not to lose the complexity of individual cases and contrast the thematic sections with full in-depth stories.

## Findings

In the interviewed men's retrospective accounts of their reproductive decision-making, surrogacy usually appeared only at a later stage of their path to parenthood. In the following sections, the ‘stages’ of a path to surrogacy are illustrated following a usual order in which they were retold by the men in this study: building up a procreative consciousness and aspirations; search for means to have children; clarifying one's motivations for surrogacy in particular; exploring the fertility industry's offer and engaging with it. Gay men's understanding of reproductive decision-making in terms of stages was also identified by other research, for example on adoptive gay fathers (Gianino, 2008, Goldberg, 2012). However, chronological and temporal approaches to ‘stages’ of reproductive decision-making have been rightly critiqued (e.g., Johnson-Hanks, 2003), not only because the multiple ‘stages’ may emerge simultaneously and overlap, which indeed happened in some of the accounts in the study presented here. The structure that stems from my data does not, then, necessarily illustrate any ‘objective’ stages of the path to surrogacy, however, it reflects the men's accounts, sense-making and reasoning about the process, elicited by my initial open-ended question, ‘How did you get to do surrogacy?’

### Changing perceptions: ‘It became more and more of an option again for me to have a child’

In their accounts, most of the interviewees distinguished what could be called the first ‘stage,’ where they developed certain reproductive aspirations or procreative consciousness at large and considered having children; and ‘stage’ two, where they contemplated specific means to become parents, including surrogacy and closer encounters with the fertility industry. Importantly, these two initial ‘stages’ in some cases overlapped at least partly.

As a starting point, the men provided diverse retrospective narratives of their motivations for parenthood. Many of them talked about their pathways to parenthood in terms of a latent potential possibility that ‘was in the background’ but not really relevant until a certain stage in their lives. Nico^6^ undertook transnational surrogacy in the USA at a specific stage following establishing his professional career at home in Germany.

It was in the background all the time, and… I can't remember, I don't think in my first gay years it was really important to me. And at that moment I always thought, well, OK, I will never be a parent, and I already had nephews and nieces so I had a lot of uncle stuff going on... It was not really that at that moment I had any parental desires. (…) I think I skipped the discussion all the time because I first wanted to be a specialist physician, it was my ambition at that moment … but gradually I also thought, ‘well, now I'm getting 33, 34, I have to do it now because otherwise it will be too late, I will be too old’. And now I'm 37 and we have a child of almost one, so that's OK for me, I think in the end it was good this way.

Nico's partner, Martin, was a few years older and already well established in his job. His desire for parenting became apparent only over time, like Nico's, and they decided to attempt surrogacy together, following a gradual process and multiple conversations.

However, a few of the other men emphasised it had always been obvious to them that they wanted to have children, as early as they could remember. They approached it as a normal pathway in life, which they often knew from their family-of-origin backgrounds. For instance Pedro, a father through transnational surrogacy for 4 months at the time of the last interview, whom I had accompanied through the surrogacy process in the USA and who was permanently residing with his child in Spain, permanently residing with his child in Spain, said he had always been ‘surrounded by a family-like environment’, himself having grown up in a big family, and later observing his own brothers and sisters having children. Pursuing parenthood seemed to him an obvious path to follow in that context. A similar story was told by Raul, whom I interviewed with his partner Vito during their trans-Atlantic visit from Italy to the USA for the birth of their surrogacy twins:

When I was younger, I had a heterosexual relationship. So with my girlfriend we talked about the possibility of having children, we were planning to get married, and then things changed, so that's another story. But I've always had a feeling that it's part of nature to have children.

Even if the men had spelled out and in some cases naturalized their motivations for parenthood so clearly, it was often interrupted by their realization of being gay, usually followed by a more or less conscious stage of ‘mourning’ over parenthood lost due to gay sexuality. It was only later that these men gradually realised that parenting could be a viable option for them, and they started to conceive of themselves as reproductive subjects. Such a gradual and reiterative process was narrated by Jelle, a father of two children through USA surrogacy, living in the Netherlands. At the moment of the interview, Jelle and his partner Tonny were expecting a third child:

When I found out I was gay, I kind of went through a mourning process in a way that I thought, OK, I'm not going to have children. But I always thought, I think, I would make a good father and I would like to have a child to take through life and show, just see them grow up, and help them to become the person that they are, it sounds kind of esoteric but that's something I always kind of felt. I did a kind of not really close the door but I went through some kind of mourning process about it. But gradually … it became more and more of an option again for me to have a child perhaps. … And then at one point we started to discuss it more explicitly. And we both wanted to look further into the options.

The men's accounts of their initial considerations of parenthood were mostly characterized by a temporal shift: first they either did not consider reproduction or they thought about it but excluded it due to being gay. However, over time they developed a kind of procreative consciousness that allowed them to see themselves both as gay and as parents. Their specific motivations ranged from considering they were at a stage in life they thought was appropriate for having children (‘I have to do it now because otherwise it will be too late’), through continuing a family sociality present in their extended kinship networks (‘I already had nephews and nieces so I had a lot of uncle stuff going on’) or even naturalized as an obvious course of events (‘I've always had a feeling that it's part of nature to have children’), up to a more individual desire to raise a child and establish a parenting relationship (‘I would like to have a child to take through life’). This early stage of reproductive decision-making, according to the men's retrospective accounts, involved developing a consciousness that rendered reproduction in their lives as gay men thinkable.

### A search for means of reproduction: ‘We wanted to have children but we didn't know how’

Reminiscing about the very beginnings of their pathways to parenthood, the men seldom referred to global fertility markets, commercial surrogacy fairs or networks. In their narratives, their early thoughts about becoming parents emerged regardless of specific options such as surrogacy, adoption or co-parenting. They often naturalised their pathways to parenthood through undisputable statements such as the above-mentioned ‘it was always in the background’ or ‘we always wanted to have children’.

Nevertheless, once these men had actually realized they wanted to have children, the dilemma many of them faced was how specifically they could do it. As expressed by Peter, who was fathering two surrogacy children with his husband Matt in California, and awaiting a third one at the time of the interview: ‘Matt and I wanted to start to do something to have a child, but we didn't know very well where to start’. Likewise, as expressed by Jonas and Marc on the other side of the Atlantic in Germany:

We always knew that we wanted to have children, but then when you turn out to be gay, and you have this different life, we just thought ‘OK, that's not gonna happen, I'm gonna be with a guy and we'll be like special uncles to our friends’ kids'. I always knew, and Marc also always knew, but... We both wanted to have children, but we didn't know how.

This last sentence was pronounced by several of the men, which evidenced that whilst they had developed some kind of procreative consciousness, they could not identify a means of reproduction straight away. In more pragmatic terms, Jason and Max, a married American couple in their mid-thirties living in California, reminisced:

‘We were much younger and we didn't have any means to do anything, of course, so it was always a sort of abstract, in the future’.

These narratives implied that becoming parents, and particularly becoming gay fathers, required finding particular means to do it. The means were understood in multiple ways: as a technique of how to actually have children (e.g. adoption, co-parenting or surrogacy); as means necessary for raising a child (such as a steady income, housing, etc. – particularly required by public adoption agencies) therefore linked to achieving a life-course stage of professional or financial stability; but also as financial means necessary to pay for surrogacy (or adoption). The men I interviewed used diverse sources to collect the approximately 120,000 to 150,000 US dollars required for a surrogacy arrangement. They mainly drew on their own earnings and savings, family help and inheritance, mortgage and other loans.

Whilst the fertility industry and surrogacy did not come up in these men's narratives about the initial emergence of their reproductive aspirations and consciousness, they did mention surrogacy with regard to their later search for specific means to achieve parenthood. In other words, the reproductive commerce was not prominent in how these men understood the emergence of their procreative consciousness at large, but it did play a part in their narratives about their specific reproductive decisions. The process of taking those reproductive decisions was interactive: when the men embarked on a search for means to have children, they needed to find information and educate themselves. In this process, two kinds of sources of information and inspiration appeared regularly. One entailed friends and acquaintances; whilst the other involved books, media and the internet, including both gay family association websites and global commercial surrogacy offerings. This showed the relevance of sociality as one of the factors in the men's reproductive decision-making. Alongside individual friends and acquaintances, fertility industries on a par with rainbow family associations became active actors within this sociality.

Vito and Raul started to consider surrogacy once they heard about it in person from friends who had done it themselves across the Atlantic. This brought home to them that surrogacy could be available to them too, contrary to the notion they had acquired from media stories that surrogacy belonged only to the world of wealthy music stars.

Vito: I ruled out the possibility of having children, because I'm gay. For me, it was just a magazine story.

Raul: It seemed impossible for us, it seemed just for Ricky Martin, for Elton John, just for rich people.

Vito: I thought it was just for rich people, just in some countries. But internally I'd always thought about children. Then, six or seven months after I met Raul, I told him about this dream and he replied, ‘I have two friends, in a town nearby, who already have twins, born in California through surrogacy, if you want we can visit them’. And we started visiting them, we listened to their story and their project. (…) So in the summer we had a vacation on an island, and I bought some books on anthropology, sociology and so on, about gay parenting.

Raul: And we started to read books, we were on a beach listening, reading books about surrogacy, about family…

Vito: And coming back from this vacation, it was August – and in October we made the first contact with the clinic, the same clinic that those friends of ours had used.

The intermediate stage of deliberation, when these men had begun exploring surrogacy but had not yet started any formal dealings with clinics, agencies or individual egg donors or surrogates, often involved reading websites and books, as well as talking to gay men who were already parents. That phase entailed exploring numerous practical considerations, understanding the usual steps and stages of a surrogacy process, and often discovering the existence of gestational surrogacy (involving both egg donors and surrogate mothers). It was particularly at that moment of reproductive decision-making that its temporal or chronological phases overlapped and boundaries between them got somewhat blurred. For some of the men, articulating their reproductive aspirations was intimately linked to imagining viable reproductive means at this stage – as for Vito and Raul, who realized they could actually have children when they met other gay men who were parents through surrogacy.

In some cases, this stage also included ‘ethical labour’ (Dow, 2016) the men did to counter their own prejudice or lack of knowledge about surrogacy. A significant part of that labour was gathering information and reasoning about ethical aspects of the process. It was particularly salient for those men who lived in countries in Europe where commercial surrogacy or surrogacy at large was not legal, and who were less familiar with it than most of the American men. Just one example came from Gerard and Pol, the fathers-of-two living in the Netherlands, who were going through their third surrogacy arrangement in the USA at the time of the interview:

We sent an email to a gay parent mailing list, asking what experiences everybody was having … And we got very negative feedback from people saying adoption was not working. We kind of thought for us surrogacy was not possible, because it's not allowed in our country so it's not an option, but then there was one gay couple who responded by saying ‘oh we're actually about to leave to the States for the birth of our twins…’ … I was very sceptical, and then I called one of the guys and I think we spoke for an hour on the phone because I was really sceptical and apprehensive about … is it ethical, is it ethically dodgy or not, and are there a lot of legal problems, and will it work, and is it very costly and is it… But in the end I was a kind of at ease with most of the things I was apprehensive about at first.

In discussing and countering their ethical dilemmas, the men were particularly apprehensive about surrogate mothers being able to take informed and relatively free decisions without threatening the men's own status as fathers. They dispelled their doubts by exploring media or personal testimonials of surrogates themselves, by conversations with the industry representatives (even if the latter may not have always represented the surrogates' and donors' voices or rights fully, see Layne, 2018, Rudrappa and Collins, 2015), and, notably, by choosing the USA as their surrogacy destination country. The established practice of commercial surrogacy in some American states, as well as the ability to interact with American surrogates in English, gave those men a sense of reassurance which, as they said, may have been more difficult to get elsewhere (for more details about the choice of the surrogacy destination country see: Smietana, 2017b).

The part of the path to surrogacy that the men retrospectively referred to as ‘searching means’ involved learning about parenting options through surrogacy, adoption and other means, including countries and specific places that provided those options. Important aspects were both personal sociality and more structured knowledge provided by marketing, the media, scholarly studies and activism. Information most often came from the men's friends, as well as sources written in books and on the internet. Also the fertility industry began to feature in the men's narratives at this stage. However, unpacking the meanings that ‘searching means’ had to the men showed that it was not only a mechanical or rational implementation of any earlier individual decisions, but rather a reiterative process where their ongoing reproductive decision-making involved gaining knowledge and reassurance that their reproductive aspirations were actually thinkable and feasible.

### Why surrogacy: ‘To have a child that can never be taken away from us’

As part of the phase of ‘searching means’ that would render their reproductive aspirations feasible, over half of the men in this study said they had considered adoption before they finally decided to opt for surrogacy. Those men did not eventually pursue adoption due to various difficulties that deterred them (e.g., evaluation and requirements on the part of adoption agencies; children's older age and their medical or other histories). On the other hand, only a few of the interviewed men discussed possibilities of co-parenting with female friends, which, however, did not work out in the end. Most of the men had rejected the option of co-parenting straight away as they thought it would be too difficult to coordinate or it would marginalize them as fathers. Mirco, a single man in his late thirties who lived in Switzerland, told me about his experience when I met him in San Francisco during his subsequent visit to meet the surrogate mother. As she was pregnant with the surrogate baby, Mirco came to accompany her at a standard ultrasound test. He recalled that before exploring surrogacy options he had first checked adoption and co-parenting.

One option was to have a child with a friend, with one of my best friends at the time. And we discussed this quite deeply, we considered this as an option … And then she found someone, so she's just had a baby with her partner right now, so I'm really happy for her, but I could see that wasn't really an option for me any more… We were talking about having a house together. I would have seen myself doing that with her but not necessarily with anybody else, ‘cause I think you know you need to have a really special kind of relationship to do this kind of things, it's not a small thing. And I have a lot of female friends, but I wouldn't just see myself having a child with them.

The men reported several motivations for choosing surrogacy over other paths to parenthood, as I discussed elsewhere (see Smietana, 2017a, Smietana, 2017b, Smietana et al., 2014). The underlying imaginary of ‘families like we'd always known’ and a desire for an ‘intact family,’ ‘our own family’ and ‘normal family’ was expressed by all of the interviewed men without exception, alongside a fear of discrimination as parents due to both their sexuality and gender identities. As pronounced by a Californian couple in their late thirties, Frank and Alex, whom I met throughout their surrogacy process:

‘So part of the discussion early on as to whether we were gonna go for something like surrogacy, part of that was to have a child that can never be taken away from us’.

Statements such as these can be understood in the context of the idealised nuclear family structure within Euro-American kinship, as well as social and legal value it renders to genetic links between fathers and children (Strathern, 1992). However, these men’s narratives must also be understood in the context of the recent histories of gay men’s marginalization and exclusion (Weeks, 2018).

### Interactions with the industry: ‘Share your family stories on Facebook and Twitter!’

In their exploration of surrogacy, the men followed on from chatting to friends and reading websites to actual conversations with the surrogacy industry representatives in the USA – whether as part of their ongoing exploration with ultimately uncertain results, or with a more determined view to conceive a baby first in a law contract and then via IVF. Most of the men in this study selected and contacted the agencies or clinics online they considered interesting given their friends' recommendations or their own criteria.^7^ This was also the case of Mirco, the single intended father from Switzerland, who had first explored adoption and co-parenting:

I didn't know anyone in person who'd have done surrogacy. So, well, I read it is common and everybody does it in the US, and I started to research the web, and I said okay why not go to the US and have a few meetings and just find out more about this, that's what I did … I found my clinic totally by chance, I would say, it was the first contact I made. Then the first trip I made here was for two weeks, and I had a lot of appointments with clinics, with agencies, with different kinds of people. Because I was not sure about the relationship I would have with the surrogate, and surrogacy may work pretty differently if you go to India or Ukraine or the US. India or the Ukraine is not gonna work for me because I'm not comfortable with the way it's done – you don't have contact with the surrogate at all, you don't meet her.^8^

Some other interviewees first attended an international fertility fair for gay intended parents, for instance an LGBTQ + parenting fair such as ‘My Future Family Show’ or a surrogacy conference organized by the association Men Having Babies. These fairs – such as the one described in the vignette opening this article – gathered together gay and reproductive activists in an alliance with fertility industry representatives, where narratives of rights intermingled with narratives of commerce.

Given that to most of the men surrogacy was initially as unknown as it was to Mirco, at least for their first surrogacy arrangements they preferred to follow the beaten track structured by agencies, clinics and legal firms, whether first contacted online or during an event in person. The intermediaries would run a matching process, whereby both intended fathers and surrogates would usually set up their online profiles and choose each other, as if quasi-dating, with a view to establishing mutual relationships and thus de-commodifying commercial surrogacy (see Berend, 2016, Jacobson, 2016, Smietana, 2017a). As the American couple Mike and Bob said, talking about Kath, their second-time surrogate at the time of the interview:

We had coffee in Starbucks about a mile from her house after work, and we talked about it... It was a very efficient conversation, like what do you care about, and we talked about religion, we talked about food, we talked about fetal reductions that we didn't want unless there was a danger to her health, we talked about, you know, like illness and things that she would control in the decision-making process, related to the health of the baby and the surrogate, what would we do in different scenarios – and I think once we aligned on those big things, then we were like ‘OK, let's contract.’

In contrast, the vast majority of commercial egg donation^9^ in the USA is anonymous; the clinics and agencies I worked with normally used anonymous egg banks, and they would welcome known donors only if intended parents brought them. Intended fathers usually choose egg donors or providers based on online profiles, using criteria such as the women's health, looks they consider attractive, education levels and artistic or other talents, and skin colour that would match their own and thus naturalize the father-child bond.^10^

Reproductive decision-making in surrogacy unfolded on throughout the entire surrogacy process. Alongside the main decision to pursue parenthood and surrogacy, numerous other decisions were involved such as choosing egg donors and surrogates, deciding which man in the couple would be the genetic father, determining potential steps in case of the need for a fetal reduction or abortion, re-evaluating the strategy in case the first embryo transfer did not work, and so on. Each one of these steps was covered by legal contracts: they could be amended by the parties before signing, whilst many of their key tenets came already structured by the industry.

Some of the men interviewed in this study also actively co-produced the stories about gay surrogacy that circulated in the media, fertility clinics and other spheres. Given they had often first heard about gay surrogacy through such stories themselves, it seems justified to argue that the gay surrogacy stories they produced could in turn further contribute to expanding other gay men's procreative consciousness.

Vito, the father from Italy I met with his partner Raul in the flat they rented in the USA while awaiting the birth of their surrogacy twins, wrote an article for a widely-read magazine in his country, with a view to demystifying surrogacy. The American couple Jeff and Daniel, whom I visited in their suburban house in California, proudly showed me a book on a central shelf in their living room published by the fertility clinic they used; the book featured their own family creation story among several others their fertility doctor put together in the volume. The photo of the fathers with their two sons was also among other family pictures hanging on the clinic walls. When I met Jeff and Daniel again at their clinic's anniversary party, we took a photo with their fertility doctor, Jack, while the doctor's daughter came up on stage to announce ‘Please share your family stories on Facebook and Twitter!’ All these spoken, written and visual stories represented a familiar mixture of publicity and community, commerce and activism, and gift and commodity exchanges, which I observed throughout this research again and again (see also Dow, 2016, Smietana, 2017a, Smietana, 2018).

### Discussion: Changing in/fertilities^11^?

In this article I explored the reproductive decision-making of 37 gay men who became fathers through surrogacy in the USA. Many of them had first decided that their homosexual orientation excluded a reproductive orientation, yet over time their perceptions of their own reproductivity gradually changed. As one of the interviewees put it, ‘it became more and more of an option again for me to have a child’. Accounts of this kind resonate with the feminist arguments that despite mainstream naturalization of reproduction as innate and self-evident, reproductive arrangements are a product of social forces and that ‘society not biology produces fertility’ (Franklin 2018: 639).

Like egg freezing in Lucy van de Wiel's analysis (Van de Wiel, 2012, Van de Wiel, 2019), surrogacy in the case of gay men is also ‘… an infertility treatment for fertile people’. Reproductive technologies such as egg freezing or surrogacy, in conjunction with the industries they represent, as well as multiple other factors, may influence people's understandings of their own fertility, as well as their perception of themselves as reproductive subjects, as has also been shown in IVF users (Franklin, 1997, Franklin, 2013).

In the interviewed men's accounts, their paths to parenthood and surrogacy were narrated as a set of temporal ‘stages’, which at times overlapped, and which began with a gradual emergence of the men's own awareness that they could have and raise children – their ‘procreative consciousness’ (Berkowitz and Marsiglio, 2007). In their accounts, only at the later stages of their paths to parenthood, when already looking for specific means of becoming parents, did they consider commercial surrogacy in the USA.

These men's paths to surrogacy also illustrated the multi-factorial character of reproductive decision-making (Taragin-Zeller, 2019, Van der Sijpt, 2014), where the narratives circulating in the men's families of origin and in the broader LGBTQ + community and media were to them of equal importance as meeting other gay men who had children, as well as reading marketing messages from surrogacy industries they came across on the internet. In the multi-faceted ontological choreography (Thompson 2005: 8) of these gay men's reproduction – alongside the reproductive intent, sperm, internet, flights, legal contracts, money, IVF, as well as couple, family and community narratives and support – a specifically new element was at play: a global surrogacy commerce, including reproductive labourers and reproductive professionals. This industry is part of a landscape within which gay men – and other populations too – are forming their perceptions of their own in/fertility today.

Yet pathways to gay fatherhood continue to be heavily stratified, and the fertility market is accessible mainly to those men who can afford commercial surrogacy in the USA (see also Jacobson, 2018, Stacey, 2006), even though some gay family associations have undertaken fundraising initiatives to mitigate the economic barriers (Men Having Babies, 2018b). It is also noteworthy that all the men I was able to recruit for this study came from the USA and Western, Northern or Southern Europe – none of them lived in post-communist Eastern Europe or other places which also testified to the ‘Western’ (Mizielinska and Stasinska, 2018) situatedness of gay surrogacy and/or queer kinships, both in terms of economics and in terms of social narratives and acceptance – even if gay men from China and Singapore have also begun commissioning surrogacy in the USA (Wang and Shan, 2017). Stratifications and situatedness of this kind must be taken into account when analysing queer kinships in the context of justice, as does the Symposium issue to which this article belongs (Smietana and Thompson, 2018).

Gay men's paths to surrogacy, and to parenthood at large, must, however, also be understood in the context of the recent and current histories of marginalization that LGBTQ + people have suffered (Briggs, 2017, Mizielinska and Stasinska, 2018, Pichardo Galán, 2011, Weeks, 2018 [1981]). In this context, to many gay people having children may mean entering mainstream family imaginaries and increased access to social inclusion. To the men in this study, having children meant a series of things alongside the children themselves: a ‘normal’ or ‘natural’ stage in life, becoming a ‘normal’ family and coalescing their couple projects, pursuing family-building paths that were normal in their extended kin networks, developing a nurturing relationship and connection to the child.

Thus gay men who become fathers through surrogacy bring queerness within the sex-gender logic where one reproduces socially by reproducing biologically. Therefore queerness cannot be readily used as a sex-gender ‘other’ in terms of which the boundaries of the heteronormative are demarcated. This disrupts the heteronormative idea that to be queer is not to contribute to the reproduction of the species, the family and the nation. These dynamics of ‘recovering fertility’ by gay men are at the heart of the consciousness change discussed in this study.

Hence this study is of relevance to current debates on queer reproductive justice (see e.g. Mamo, 2018, Stacey, 2018). Given that reproductive decision-making is taking place in stratified contexts, and queer people – in particular gay men – have been excluded from mainstream family imaginaries and collective ‘procreative consciousness’, a perspective of justice could now posit that gay men gain conditions to build procreative consciousness and receive support for making families. A novel example of such support is state-funded IVF to be used in gestational surrogacy, for the first time given to a British gay couple by Scottish National Health Fund in January 2019 (Braidwood, 2019). On the other hand, the perspective of justice requires that gay men's reproductive decision-making does not take precedence over the well-being and decisions of surrogate mothers and egg donors (see Gunnarsson Payne, 2018 this volume; Smietana et al., 2018 this volume) – or over the well-being of other species and the planet (Sturgeon, 2010).

## Funding sources

This research was funded the British Wellcome Trust (grant no. 209829/Z/17/Z, and grant no. 100606), as well as by the European Commission’s Marie Skłodowska-Curie International Outgoing Fellowship (FP7-PEOPLE-2013-IOF, Grant Agreement no. 629341, ‘SurrogARTs’ project). Complementary funding was also provided by the Spanish Ministry of Economy, Industry and Competitiveness (grant no. CSO2015-64551-C3-1-R).

## Footnotes

1 I use the term ‘gay’ mainly with regard to individuals who self-identify as non-heterosexual men. Where indicated, I also use it in a broader meaning with regard to non-heterosexual people of any sex or gender, e.g. ‘gay people’ – in this broad meaning, I use the terms ‘gay,’ ‘non-heterosexual,’ ‘queer’ and ‘LGBTQ +’ interchangeably, unless indicated otherwise. For the complex and often divergent histories and uses of these terms in the context of making families and kinship, in particular ‘LGBTQ +’ or ‘gay’ as opposed to ‘queer’ and also ‘trans,’ please see Smietana et al., 2018, this volume.

2 I use the terms ‘fertility industry,’ ‘reproductive commerce,’ and ‘reproductive market’ interchangeably, given the limited space and the article's main focus on reproductive decision-making, of which the fertility industry is only one aspect. By ‘fertility industry’ I refer to clinics, agencies, bio-banks and other infrastructure, as well as exchanges and arrangements linked to them, through which commercial surrogacy in the United States is organized, provided and marketed. For more detailed analyses of the USA fertility and surrogacy industry see e.g. Thompson, 2005, Thompson, 2016, Spar and Harrington, 2009, Jacobson, 2016.

3 For more details please see: www.surrogarts.eu, www.reprosoc.sociology.cam.ac.uk/projects/surrogarts

4 I use the terms ‘Europe’ and ‘European’ to refer to the interviewees' countries of residence, for which the common denominator was that those countries belonged to the European Economic Community at the time of this research (apart from Switzerland which belonged to the European single market). Although this article uses the lens of surrogacy carried out by all the men in the USA, it must be recognized that the legal, cultural and other local specificities in each one of the countries loosely termed here ‘European’ may have influenced the perceptions and experiences of surrogacy significantly, as shown e.g. by Jérôme Courduriès (2018 this volume) in case of France.

5 The study was approved by the Committee for Protection of Human Subjects at the University of California-Berkeley, protocol # 2014-10-6793.

6 All interviewees' names are pseudonyms.

7 The present study did not include a systematic analysis of the online information the interviewed men interacted with. For such analyses, see e.g. Jacobson, 2018 this volume; Stuvøy, 2018, Whittaker, 2019.

8 Forging friendship relationships with surrogates, as well as the established surrogacy practice and laws in the USA (in particular California and Oregon where this study was carried out) were for the interviewed men some of the important reasons why they chose the USA as their surrogacy destination – apart from the fact they could afford it, given surrogacy costs in the USA are higher than in other countries. For more details on the choice of the surrogacy destination country see Smietana, 2017b.

9 All the men in this study entered gestational surrogacy arrangements, which involve both a surrogate who carries the baby and an egg donor who provides the genetic material. Gestational surrogacy is currently promoted by the USA fertility industry, as opposed to traditional surrogacy where the surrogate uses her own genetic material.

10 Despite these inclusive intentions that the parents often have, matching parents and donors’ skin colour may contribute to the reproduction of notions such as ‘racial purity’. It may also contribute to the reproduction of white supremacy, given the racialized character of the fertility industry, whose vast majority of customers are of white skin colour. For more detailed analyses please see Roberts, 1997, Thompson, 2009; and Twine, 2017.

11 I owe my gratitude for this term to Prof. Sarah Franklin and Prof. Marcia Inhorn, Principal Investigators of the ‘Changing In/Fertilities’ project, carried out within the Wellcome Trust Collaborative Award (2018–2021, grant no. 209829/Z/17/Z) at the Reproductive Sociology Research Group, University of Cambridge. The term ‘changing in/fertilities’ refers to the ongoing shifts in perceptions of fertility and reproduction, as well as changing reproductive behaviours.
